# Temperature-Adaptive Activation Energy for Maturity-Based Strength Prediction of Sustainable, SCM-Blended Self-Compacting Concrete

**DOI:** 10.3390/ma19163462

**Published:** 2026-08-14

**Authors:** Abdulaziz Aldawish, Sivakumar Kulasegaram, Ayman Almutlaqah, Abdullah Alshahrani

**Affiliations:** 1 School of Engineering, Cardiff University, Cardiff CF24 3AA, UK; kulasegarams@cardiff.ac.uk; 2 College of Engineering and Energy, Abdullah Al Salem University, Khaldiya 72303, Kuwait; 3 Department of Civil Engineering, College of Engineering, Najran University, Najran 61441, Saudi Arabia; aaalmutlaqa@nu.edu.sa (A.A.); aaalshahranie@nu.edu.sa (A.A.); 4 Science and Engineering Research Center, Najran University, Najran 61441, Saudi Arabia

**Keywords:** self-compacting concrete, maturity method, apparent activation energy, supplementary cementitious materials, compressive strength prediction, hydration kinetics, temperature-dependent activation energy

## Abstract

**Highlights:**

- Temperature-adaptive *Q*(*T*) extends the ASTM C1074 maturity method to SCM-blended SCC.
- β_Q_ is a signed binder fingerprint: significantly negative for the FA, GGBS and RHA systems.
- The linear *Q*(*T*) model achieves *R*^2^ = 0.929 (RMSE = 4.74 MPa) on 196 SCC observations (five-fold cross-validation: *R*^2^ = 0.901).
- One added parameter removes the constant-energy model’s temperature bias, confirmed by AIC/BIC.
- External validation on 120 literature observations gives *R*^2^ = 0.881 with near-zero bias.

**Abstract:**

The maturity method (ASTM C1074) predicts in situ concrete strength from a recorded temperature history but assumes a constant apparent activation energy, contradicting the experimental evidence that the activation energy falls as hydration shifts from kinetics control to diffusion control—an effect that differs between binder chemistries when supplementary cementitious materials (SCMs) are used. This study develops a physics-based maturity model in which the apparent activation energy varies linearly with temperature, *Q*(*T*) = *Q*_0_ + β_Q_(*T* − *T*_ref_), coupling a variable-energy Arrhenius equivalent age to a hyperbolic strength–maturity relationship. The model was calibrated on 196 mean-strength observations (588 cube tests) from seven self-compacting concrete mixtures cured isothermally at 10, 20, 35 and 50 °C and tested at seven ages (1–90 days). All four SCM systems (fly ash, GGBS, silica fume and rice husk ash) returned a negative coefficient (−210 to −974), enclosing the temperature sensitivity implied by independent calorimetric measurements on Portland cement paste (≈−580 J/(mol·K)), whereas the ordinary Portland cement control returned a positive point estimate (+101) that is not statistically distinguishable from zero. The model achieved *R*^2^ = 0.929 (RMSE = 4.74 MPa), outperforming the constant-energy ASTM C1074 baseline in both accuracy and the Akaike Information Criterion while eliminating its systematic bias at the temperature extremes. Five-fold cross-validation confirms the out-of-sample accuracy (*R*^2^ = 0.901, RMSE = 5.59 MPa), and bootstrap analysis shows the negative coefficients of the fly ash, GGBS and rice husk ash systems to be statistically significant. External validation on 120 independent literature observations gave *R*^2^ = 0.881.

## 1. Introduction

Accurate in situ strength prediction underpins critical construction decisions, including formwork and falsework removal, post-tensioning, and load application, in temperature-sensitive concreting, where relying on standard-cured specimens can misrepresent the strength of concrete that has experienced a different thermal history. The maturity method addresses this need through the principle that the strength gain of a given concrete is governed jointly by its age and its curing temperature, which can be combined into a single index—the maturity—that maps uniquely to the strength of that mixture. In practice the temperature history recorded by an embedded sensor is converted into a maturity index, and strength is then read from a strength–maturity relationship calibrated once for the mixture, enabling non-destructive, real-time estimation of in-place strength.

Two formulations dominate the calculation of the maturity index from a recorded temperature history. The Nurse–Saul temperature–time factor assumes the rate of strength gain varies linearly with temperature; the Arrhenius-based equivalent age assumes an exponential (Arrhenius) rate–temperature dependence and converts the thermal history into an equivalent duration of curing at a reference temperature (typically 20 °C). The Arrhenius form is generally the more accurate of the two across the wide temperature ranges encountered in cold- and hot-weather placement, and both are standardised in ASTM C1074 [1]. Because the equivalent-age calculation hinges on the apparent activation energy denoted here as *Q*, the value assigned to *Q* directly influences the predicted strength—making the estimation of *Q* the central modelling choice in any Arrhenius maturity implementation, and is thus the focus of this study.

The conventional ASTM C1074 implementation assumes a single constant apparent activation energy *Q* across all temperatures. This assumption contradicts well-established experimental evidence: the rate-limiting mechanism of cement hydration changes with temperature; hence, *Q* is not constant. At low temperatures (<15 °C), hydration is kinetics-controlled (nucleation and growth of calcium silicate hydrate (C-S-H) gel), with high *Q* ≈ 40–60 kJ/mol; at high temperatures (>35 °C), it becomes diffusion-controlled (ion transport through dense C-S-H), with low *Q* ≈ 20–35 kJ/mol. This mechanistic crossover—documented calorimetrically by Kjellsen and Detwiler [2] and modelled by Schindler [3]—causes a constant-*Q* model to systematically overpredict strength at low temperatures and underpredict it at high temperatures, introducing directional bias precisely where formwork-removal decisions are most timing-critical.

The challenge deepens when supplementary cementitious materials are used. Supplementary cementitious materials (SCMs) react through pozzolanic or latent-hydraulic mechanisms that differ from the hydration process of Portland cement. As a result, SCM-blended systems exhibit activation energies that are distinct from those of plain ordinary Portland cement (OPC), reflecting differences in reaction pathways and temperature sensitivity [4,5,6]. In addition, the mechanisms governing hydration and strength development in SCM-blended systems evolve with curing conditions and the degree of hydration, leading to variations in apparent activation energy over time. Consequently, the temperature-dependent strength development of SCM-containing concretes differs from that of conventional Portland cement systems, highlighting the importance of accounting for SCM effects in maturity-based models [2,3]. This challenge is particularly significant for self-compacting concrete (SCC), where the high powder content, low water-to-binder ratio, and extensive use of superplasticizers alter hydration kinetics, pore structure evolution, and microstructural development compared with conventional vibrated concrete [7,8]. As a result, the applicability and accuracy of conventional maturity models may be limited when applied to SCC mixtures incorporating high volumes of SCMs.

**Figure 1:**
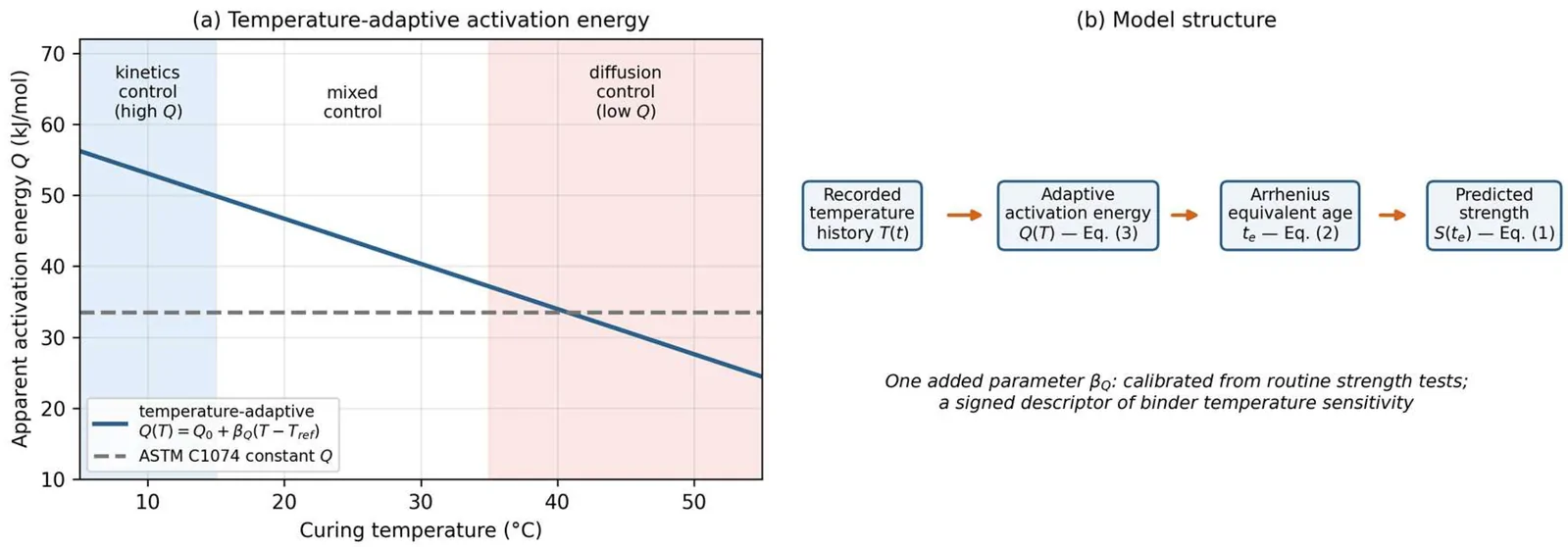
Concept of the temperature-adaptive maturity model: (**a**) the apparent activation energy declines from the kinetics-controlled to the diffusion-controlled regime and is represented by the linear law *Q*(*T*) = *Q*_0_ + β_Q_(*T* − *T*_ref_), in contrast to the constant-*Q* assumption of ASTM C1074 [1]; (**b**) structure of the model chain, Equations (1)–(3).

This paper develops a physics-based maturity model whose central contribution is not merely to improve accuracy but also to provide an interpretable parameter that characterises the binder’s temperature response. In the proposed approach, the apparent activation energy is allowed to vary with temperature through a single added parameter, a temperature-sensitivity coefficient that is recovered directly from routine compressive-strength measurements rather than from isothermal calorimetry. Its sign and magnitude are interpreted as a binder fingerprint that distinguishes SCM-blended systems from the plain OPC control mix. The full formulation, including the governing equations, is developed in Section 2.2 (Figure 1). The relationship of the present study to the authors’ earlier work should be stated explicitly. The experimental strength dataset was generated and first reported in Ref. [9], and a companion study [10] applied the standard fib Model Code maturity function, with its fixed temperature law, to the same data. Everything else reported here is new: the temperature-adaptive activation-energy law and its coupling to the maturity framework, the calibrated binder fingerprints, the information-criterion model comparison, the cross-validation and bootstrap uncertainty analyses, and the external validation on independent literature data appear in neither previous publication.

In summary, this study aims to: (i) develop a physics-based maturity model in which the apparent activation energy is coupled with temperature through a single interpretable parameter, the temperature-sensitivity coefficient; (ii) calibrate the model on a controlled laboratory dataset of seven SCC mixtures spanning four curing temperatures (10–50 °C) and seven different ages (1–90 days); (iii) demonstrate that the calibrated temperature-sensitivity coefficient is physically coherent and quantitatively consistent with independent calorimetric data; (iv) establish, using the Akaike and Bayesian Information Criteria, how much activation-energy complexity the data actually support relative to the constant-*Q* ASTM C1074 baseline and a quadratic alternative; (v) validate the model on independent literature data; and (vi) quantify the out-of-sample predictive accuracy and the parameter uncertainty through cross-validation and bootstrap resampling. The overarching objective is a parsimonious, interpretable alternative to constant-*Q* maturity practice tailored to the SCM-blended mixtures increasingly specified for low-carbon construction. Reducing embodied carbon has, moreover, become a central criterion in structural material selection—within circular-economy strategies for the built environment [11], in comparative embodied-carbon assessments of superstructure framing systems [12], and in the environmental optimisation of foundation elements such as piles [13]. Within concrete construction, partial replacement of clinker by SCMs is the principal near-term decarbonisation lever, so reliable strength prediction for SCM-blended mixtures directly supports these material-selection decisions.

## 2. Materials and Methods

### 2.1. Laboratory Dataset

The laboratory dataset was designed to calibrate the temperature-adaptive maturity model across a wide temperature (10–50 °C) and age (1–90 days) envelope. Seven high-strength SCC mixtures were cured isothermally at four target temperatures (10, 20, 35, and 50 °C) in temperature-controlled water tanks and tested at seven ages (1, 3, 7, 14, 28, 56, and 90 days) as per BS EN 12390-3 [14]. At each mixture–temperature–age combination, three replicate 100 × 100 × 100 mm cubes were tested and their mean recorded as the representative strength. The full experimental programme—mixture proportions, fresh-state properties, and the complete strength dataset—was reported previously [9]; the consolidated strengths are re-analysed here for maturity modelling. The four curing temperatures (10–50 °C) span the practical field envelope from cold-weather concreting to hot-weather and steam-cured precast placement, while the seven test ages (1–90 days) provide dense early-age sampling for formwork-removal decisions together with sufficient late-age coverage to characterise the limiting strength and the slow pozzolanic contribution of the SCM systems. The seven mixtures comprise a plain OPC control mix together with six SCM blends including fly ash (FA), ground granulated blast-furnace slag (GGBS), silica fume (SF) and rice husk ash (RHA) at the replacement levels indicated by their mixture codes (e.g., SCC-FA-20 denotes 20% replacement of cement by fly ash, and SCC-GGBS-40 denotes 40% replacement by GGBS). The dataset comprises 196 consolidated mean-strength observations, equivalent to 588 individual cube tests, every mixture following the same full 4 × 7 (temperature × age) design and therefore contributing 28 consolidated observations.

Because all specimens were cured in temperature-controlled water tanks, they remained continuously saturated, so differences in strength can be attributed to temperature and age alone rather than to moisture loss. The coefficient of variation among the three replicate cubes tested at each mixture–temperature–age condition averaged 4.9% across the dataset; the larger values were confined to early ages and low curing temperatures, where the small absolute strengths inflate the percentage scatter.

Table 1 summarises compressive strength by material system, with each mean pooled across all four curing temperatures and seven ages.

**Figure 2:**
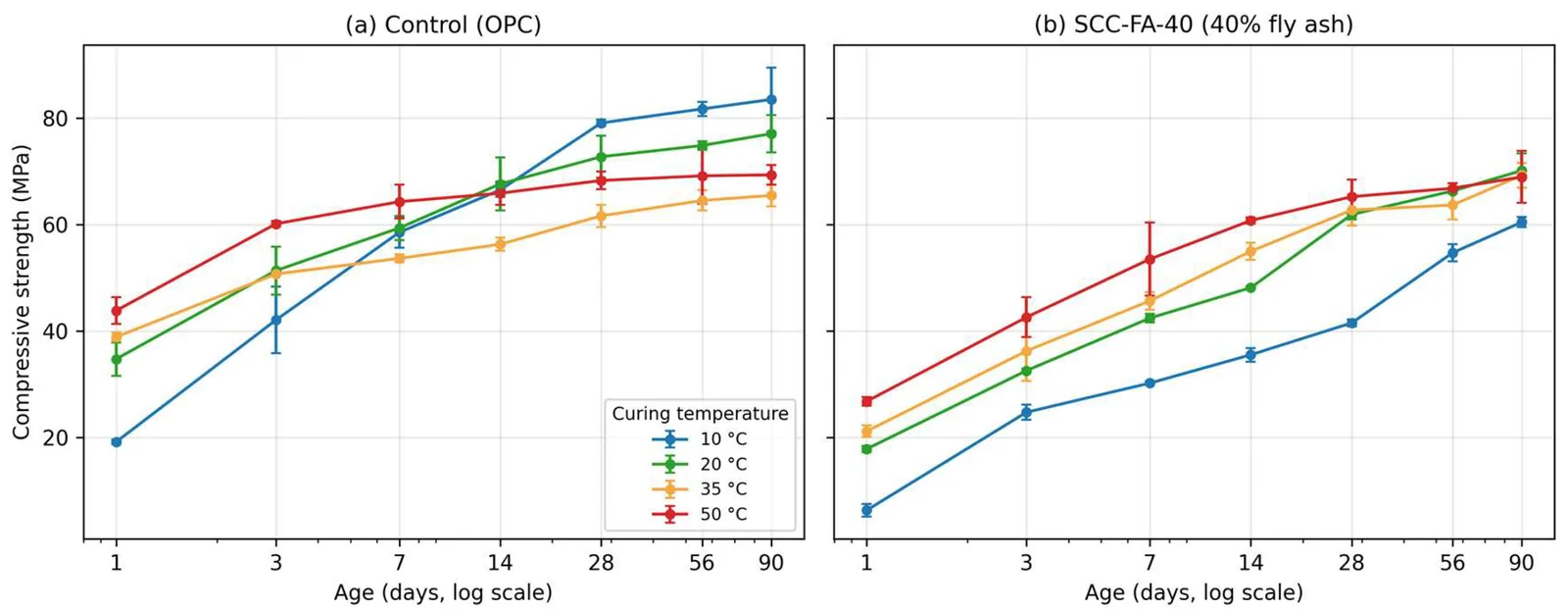
Compressive-strength development at the four isothermal curing temperatures: (**a**) Control (OPC) mixture; (**b**) SCC-FA-40. Error bars show ± one standard deviation of the three replicate cubes. Data from [9].

The temperature–strength behaviour directly motivates the model and is documented in full in the dataset found in [9]. Corresponding strength development of the Control mixture is shown in Figure 2. At early ages (1–3 days), strength increases monotonically with curing temperature, as expected from Arrhenius acceleration; by 14 days the order partially reverses, with specimens cured at 10 and 20 °C overtaking those cured at 35 and 50 °C (Figure 2). The standard explanation is that high-temperature curing produces a coarser pore structure through rapid C-S-H precipitation, lowering the limiting strength [2,15]. The early-age temperature sensitivity is far more pronounced for SCC-FA-40 than for the Control mix (Figure 2b). At low temperature the pozzolanic reaction of fly ash is strongly retarded, so the early-age strength of SCC-FA-40 relies almost entirely on the diluted cement fraction—reflected in the dataset minimum of 6.4 MPa, recorded at 1 day under 10 °C curing and the lowest strength among all temperature–age combinations listed in Table 1—whereas elevated temperature accelerates the pozzolanic reaction disproportionately and boosts early strength [10]. At later ages this advantage reverses: the hydrates formed rapidly at high temperature create a coarser pore structure with a lower limiting strength [2,15], while the slowly reacting fly ash at low temperature continues to refine the microstructure and to gain strength up to 90 days. Within the 90-day test window, this produces only a partial reversal for SCC-FA-40: by 90 days, the 20 °C specimens overtake those cured at 50 °C while the 10 °C specimens are still gaining strength steeply, whereas the reversal of the Control mix is complete from 28 days (Figure 2). The pozzolanic reaction thus extends the strength-development window at low temperature, and the two panels of Figure 2 contrast these behaviours directly.

### 2.2. Model Formulation

The proposed temperature-adaptive maturity model comprises three coupled equations: a strength–maturity relationship, an Arrhenius equivalent age, and the temperature-adaptive activation-energy law which is the central contribution of this work. The first is the hyperbolic strength–maturity relationship introduced by Carino [16] and adopted in ASTM C1074 [1], in which strength approaches a limiting value asymptotically as a function of equivalent age:

(1) $$S\left(t_{e}\right)=\frac{\left(S_{u0}-\lambda r\right)kt_{e}}{1+kt_{e}}$$

where *S*(*t*_e_) is the compressive strength (MPa) at equivalent age *t*_e_; *S*_u0_ is the ultimate-strength parameter (MPa) for the OPC control mix as *t*_e_ → ∞; λ is the strength-reduction coefficient (MPa) per unit SCM replacement ratio; *r* is the SCM replacement ratio (0–1, dimensionless); *k* is the rate constant (day^−1^) governing early-age strength gain; and *t*_e_ is the equivalent age (days) at reference temperature *T*_ref_ = 20 °C. The hyperbolic form follows from Powers’ gel–space ratio theory [17], in which compressive strength is proportional to the volume fraction of hydration products, so that strength rises rapidly at early ages and approaches the ultimate value *S*_u_ = *S*_u0_ − λ*r* asymptotically as hydration nears completion. The standard single-asymptote form is extended here by expressing the ultimate strength as *S*_u0_ − λ*r*, allowing the limiting strength to decrease linearly with SCM replacement ratio *r* and thereby capturing the dilution effect of cement replacement within a single relationship.

The second equation is the Arrhenius equivalent age:

(2) $$t_{e}=\int_{0}^{t}\exp\left[-\frac{Q\left(T\right)}{R}\left(\frac{1}{T}-\frac{1}{T_{ref}}\right)\right]dt^{\prime }$$

where *Q*(*T*) is the temperature-adaptive apparent activation energy (J/mol), *R* = 8.314 J/(mol·K) is the universal gas constant, *T* is the absolute curing temperature (K), and *T*_ref_ = 293 K; t′ denotes the time variable of integration. For the isothermal curing used here the temperature is constant, so the integrand in Equation (2) is time-invariant and the equivalent age reduces to the product of the elapsed time and the constant Arrhenius factor evaluated at the curing temperature, while the integral form accommodates the non-isothermal histories of field placements.

The third equation is the temperature-adaptive activation energy, and it carries the focus of this paper:

(3) $$Q\left(T\right)=Q_{0}+\beta_{Q}\left(T-T_{ref}\right)$$

where *Q*_0_ is the baseline activation energy (J/mol) at *T*_ref_ and β_Q_ is the temperature-sensitivity coefficient (J/(mol·K)). The above form in Equation (3) is a first-order Taylor expansion of an unknown apparent activation-energy function *Q*(*T*) about the reference temperature *T*_ref_, retaining only the leading temperature-dependent term. This choice is motivated by three key factors. First, mechanistically: the rate-controlling step of hydration shifts continuously from reaction control at low temperature (high *Q*) to diffusion control at high temperature (low *Q*) [2,3], a monotonic transition that a linear term in *T* captures to first order without piecewise logic or regime-detection thresholds. Second, empirically: published calorimetric measurements of *Q* across temperature for cementitious systems are well approximated by a linear decline over the practical curing range. Therefore, a linear law is consistent with the measured behaviour rather than one imposed on it. Third, on parsimony: the linear form adds exactly one parameter, β_Q_, to the constant-*Q* ASTM C1074 baseline (recovered as the special case β_Q_ = 0), making the model the minimal extension that introduces temperature dependence. Section 3.4 tests this choice directly by comparing the linear law against both the constant-*Q* baseline and a quadratic extension using information criteria and shows that the data support precisely one degree of temperature freedom—neither fewer nor more.

**Figure 3:**
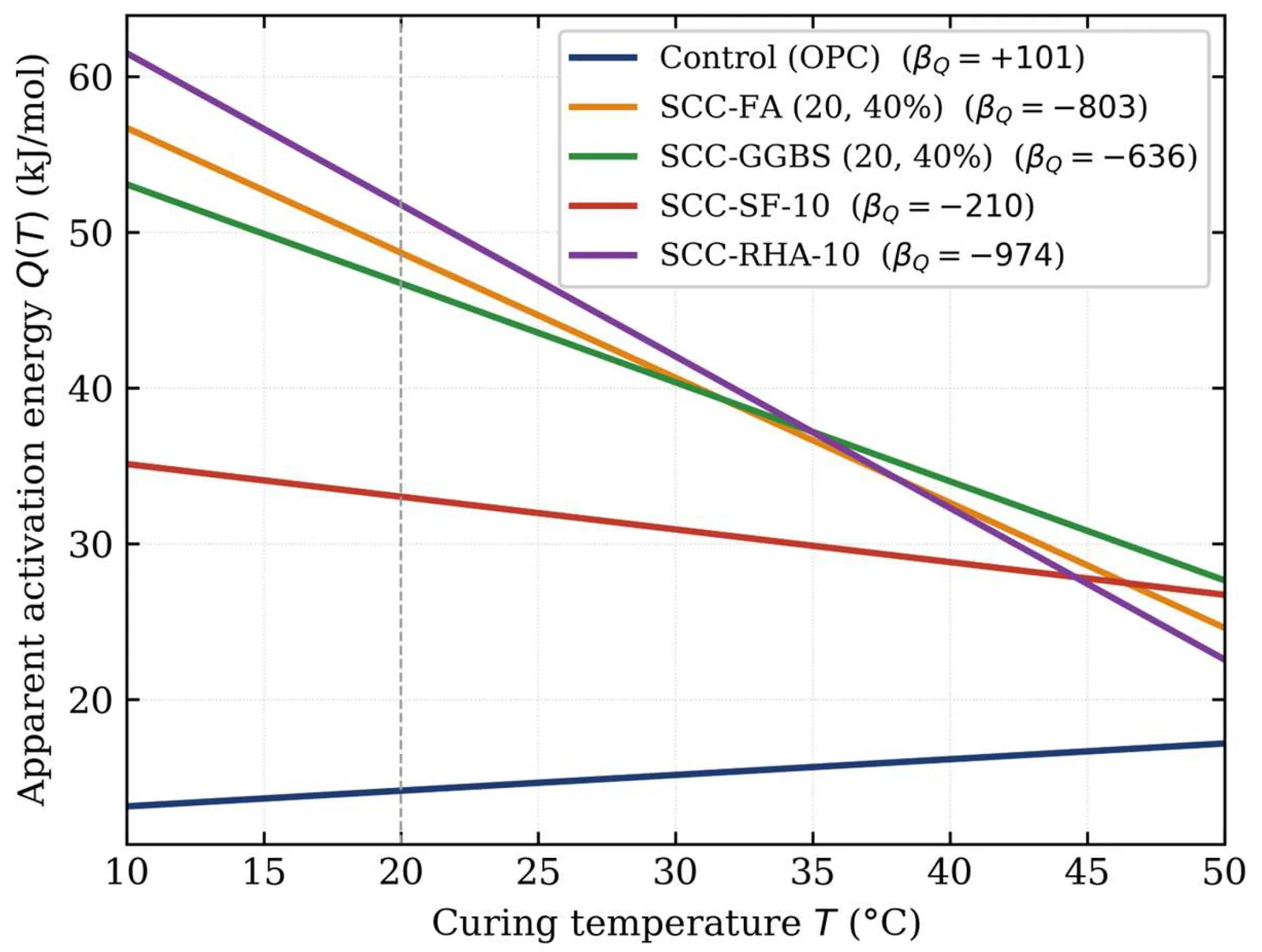
Calibrated temperature-adaptive activation-energy functions Q(T) = Q_0_ + β_Q_(T − T_ref_) for the five binder systems over the experimental range; the vertical dashed line marks the reference temperature T_ref_ = 20 °C (10–50 °C).

This linear form distinguishes the proposed approach from the conventional ASTM C1074 baseline, which fixes β_Q_ = 0. It interpolates smoothly across the mixed-control range (i.e., curing temperatures of approximately 15–35 °C) between the kinetics-controlled regime at low temperature (high *Q*, ≈45–60 kJ/mol) and the diffusion-controlled regime at high temperature (low *Q*, ≈20–35 kJ/mol), without piecewise logic or regime detection (Figure 3). Crucially, β_Q_ is not a random fitting parameter but a physical descriptor: its sign reports the direction of the dominant temperature effect—negative when the rate-limiting step shifts from reaction to diffusion as temperature rises (expected for SCM systems), positive when the late-age crossover dominates (possible for the OPC control mix)—and its magnitude grades how strongly that effect operates.

Table 2 lists the five model parameters and their calibration bounds, informed by the hydration-kinetics literature [3,4,5] and by exploratory analysis of the dataset. The asymmetric bounds on β_Q_ reflect the expectation of predominantly negative values while admitting the positive OPC-related crossover. The numerical limits were chosen to be deliberately wide: the lower bound of −1000 J/(mol·K) comfortably accommodates strongly negative temperature sensitivities, while the upper bound of +500 J/(mol·K) admits a positive slope should the crossover effect dominate. Exploratory fits confirmed that all calibrated values lie well inside these limits.

### 2.3. Calibration Procedure

The five model parameters listed in Table 2 were estimated by minimising the sum of squared errors (SSE) between measured and predicted strengths,

(4) $$\underset{\theta }{\min}SSE\left(\theta \right)=\sum_{i=1}^{N}{\left(y_{i}-{\hat{y}}_{i}\left(\theta \right)\right)}^{2}$$

by using a two-stage strategy. In Stage 1, a stochastic, population-based Differential Evolution (DE) algorithm explores the five-dimensional parameter space to locate the region of the global optimum; it used a population size of 20 times the number of parameters, mutation factor *F* = 0.8, crossover probability *CR* = 0.7, a maximum of 1000 iterations, and a convergence tolerance of 10^−7^. In Stage 2, the best DE solution seeds a bounded L-BFGS-B (Limited-memory Broyden–Fletcher–Goldfarb–Shanno with box constraints) refinement, with finite-difference gradients. The DE stage was chosen because the five-parameter error surface is non-convex, and a gradient-free, population-based global search avoids any dependence on starting values; the population is initialised by Latin-hypercube sampling within the bounds of Table 2 (the scipy.optimize implementation was used (SciPy version 1.17, Python 3.12)), and the search terminates when the relative spread of the population energies falls below the stated tolerance or the iteration cap is reached. The two-stage design assigns complementary roles: DE locates the basin of the global optimum and L-BFGS-B refines the solution within it. In practice the local stage changed the DE solution only marginally, and repeating the entire calibration from four independent random initialisations reproduced the optimal error of every material group to within numerical precision, confirming that the estimates are insensitive to initialisation. As a further check of reproducibility, an independent re-implementation of the complete procedure, prepared during revision, reproduced every calibrated parameter (Section 3.1) and the overall *R*^2^ = 0.929 exactly. The FA and GGBS families were fitted jointly across their two replacement levels—that is, the 28 observations of each of FA-20 and FA-40 were pooled into a single 56-observation optimisation that returns one parameter set for the fly-ash binder, and likewise for GGBS-20 and GGBS-40—(sharing *S*_u0_, λ, *k*, *Q*_0_, and β_Q_) because the apparent activation energy is governed predominantly by the binder chemistry, with replacement level a secondary effect [4,5], with per-mixture metrics evaluated separately on each subset; the OPC control mix uses four parameters (λ being inapplicable at *r* = 0). The same procedure and group structure were applied to the benchmark variants (constant-*Q* and quadratic *Q*(*T*)), so that the variants differ only in the functional form of *Q*(*T*) and the parameter count.

### 2.4. Cross-Validation, Uncertainty Quantification and External-Validation Protocol

Three complementary out-of-sample protocols quantify predictive rather than in-sample accuracy, following the strict separation of calibration and assessment data advocated for predictive strength models [18]. (i) Five-fold cross-validation: The 196 observations were partitioned into five folds, stratified within each mixture; every material group was recalibrated on four folds with the full two-stage procedure and evaluated on the held-out fold, and the pooled held-out predictions are reported. (ii) Leave-one-temperature-out validation: All 49 observations at one curing temperature were withheld, the model was recalibrated on the remaining three temperatures and the withheld temperature was predicted—an intentionally severe test of temperature transfer that involves extrapolation of *Q*(*T*) whenever the 10 or 50 °C set is withheld. (iii) Leave-one-replacement-level-out validation for the FA and GGBS families: The model calibrated on one replacement level predicts the other through the dilution term λ*r*.

Parameter uncertainty was quantified by a parametric bootstrap that propagates the measured replicate variability directly: in each of B = 500 resamples, every group mean was perturbed by a normal deviate with the standard error of its three replicate cubes, and all parameters were re-estimated by bounded L-BFGS-B started from the full-data optimum. Percentile 95% confidence intervals, parameter correlations and 95% prediction intervals combining parameter and residual uncertainty are reported in Section 3.8. Confidence intervals for the external-validation subsets (Section 3.5) were obtained by case resampling of the external observations (2000 draws).

## 3. Results

### 3.1. Calibrated Parameters

Table 3 presents the calibrated parameters for all seven SCC material systems; entries shared between FA-20/FA-40 and between GGBS-20/GGBS-40 reflect the joint family fits described in Section 2.3, with R^2^ and RMSE evaluated separately for each mixture. The ultimate-strength parameter *S*_u0_ is highest for SCC-SF-10 (82.81 MPa); the Control sits at the baseline 72.65 MPa; and the FA family’s shared *S*_u0_ = 80.48 MPa reflects the joint fit across two replacement levels. The dilution coefficient λ varies the most: FA shows a high λ = 40.28 MPa (implying ≈ 16.1 and 8.1 MPa reductions at 40% and 20% replacement, respectively) and GGBS a moderate λ = 16.18 MPa, while the SF and RHA values—each fitted from a single replacement level—are weakly identified and should be interpreted with caution: with data at a single replacement level, λ and *S*_u0_ cannot be separated—only their combination *S*_u0_ − λ*r* is constrained by the data—so the fitted λ describes the asymptotic strength of that particular mixture and should not be extrapolated to other replacement levels. The rate constant *k* is highest for the Control mix (0.681 day^−1^) and lowest for FA (0.323 day^−1^), consistent with the delayed pozzolanic reaction of fly ash.

The temperature sensitivity coefficient β_Q_ carries the central parameter of the model. All four SCM systems return a negative β_Q_, −210 J/(mol·K) for SF, −636 for GGBS, −803 for FA, and −974 for RHA, confirming the kinetics-to-diffusion transition hypothesis, while the OPC control mix alone returns a positive point estimate of β_Q_ = +101 J/(mol·K), consistent with the crossover effect (see Section 3.8 for its uncertainty). Every calibrated value lies within the interior of the search interval, so that the ordering of the magnitudes (|β_Q_| increasing from SF through GGBS and FA to RHA) is determined by the data rather than by the bounds. Figure 3 illustrates the calibrated *Q*(*T*) functions: the four SCM curves are most widely separated at 10 °C (*Q* ≈ 35–62 kJ/mol), where binder differences are largest, and converge to a narrow band (≈22–28 kJ/mol) toward 50 °C, where diffusion control dominates regardless of binder chemistry, while the OPC control mix line lies below the SCM curves throughout and rises gently with temperature, reflecting its small positive β_Q_. Because the apparent activation energy is governed predominantly by the binder chemistry, with the cement-replacement level only a secondary influence [4,5], the two fly-ash mixtures (FA-20, FA-40) share a single *Q*(*T*) function, as do the two GGBS mixtures (GGBS-20, GGBS-40); the replacement level enters the model only through the strength-dilution term λ*r* in Equation (1).

### 3.2. β_Q_ as a Signed Binder Fingerprint

The defining result of this study is that the single parameter β_Q_ discriminates binder chemistry by sign and magnitude, recovering distinctions that normally require calorimetry. Its sign clearly separates two mechanisms of thermal kinetics. The negative β_Q_ of every SCM system is consistent with a kinetics-to-diffusion transition. At low temperature (10 °C), hydration is kinetics-controlled, with a high *Q* reflecting the large energy barrier for breaking Si–O and Ca–O bonds in SCM particles; the pozzolanic reaction is very slow and strength development is dominated by residual cement hydration [10]. At moderate temperature (20–35 °C), mixed control operates and *Q* falls linearly. At high temperature (50 °C), hydration becomes diffusion-controlled: the pozzolanic reaction is rapid, but the coarse microstructure from fast C-S-H precipitation reduces ultimate strength, and the low *Q* reflects the smaller barrier for ion diffusion than for chemical reaction. The positive β_Q_ = +101 J/(mol·K) of the OPC control mix is a point estimate consistent with the crossover phenomenon [15], although its bootstrap confidence interval spans zero (Section 3.8), so this reading is indicative rather than established: a constant-*Q* model cannot capture the partial reversal of the strength order at late ages, and the temperature-adaptive *Q*(*T*) supplies an empirical correction in which a higher effective *Q* at high *T* slows equivalent-age accumulation, partially offsetting rapid early-age hydration.

The relative magnitudes of β_Q_ rank the SCMs in a way that is physically meaningful. SF is the least temperature-sensitive (β_Q_ = −210), consistent with its highly reactive, fine pozzolan that reacts substantially even at low temperature, so the rate-limiting mechanism changes least with temperature. FA and RHA are the most temperature-sensitive (−803 and −974), consistent with sluggish low-temperature pozzolanic reactions that accelerate sharply on heating. GGBS sits between (−636), in line with its latent-hydraulic character, intermediate between the strong pozzolan SF and the slow pozzolans FA and RHA.

These calibrated values are quantitatively consistent with independent calorimetric data, which supports the interpretation that β_Q_ reflects a genuine characteristic of the binder chemistry for these systems; for OPC and SF the sign is indicative only. The negative coefficients obtained here for the SCM systems are consistent with the established decline of apparent activation energy as hydration shifts from reaction control to diffusion control [2,3], indicating that β_Q_ captures the underlying hydration kinetics rather than artefacts of the fitting procedure, and supports its use as a compact, transferable descriptor of binder behaviour. Because the apparent activation energy characterises the hydration kinetics of the binder itself, it is routinely transferred between paste, mortar and concrete—ASTM C1074 determines *Q* from mortar specimens [1]—so estimating it here from concrete strength data is appropriate.

### 3.3. Predictive Performance

**Figure 4:**
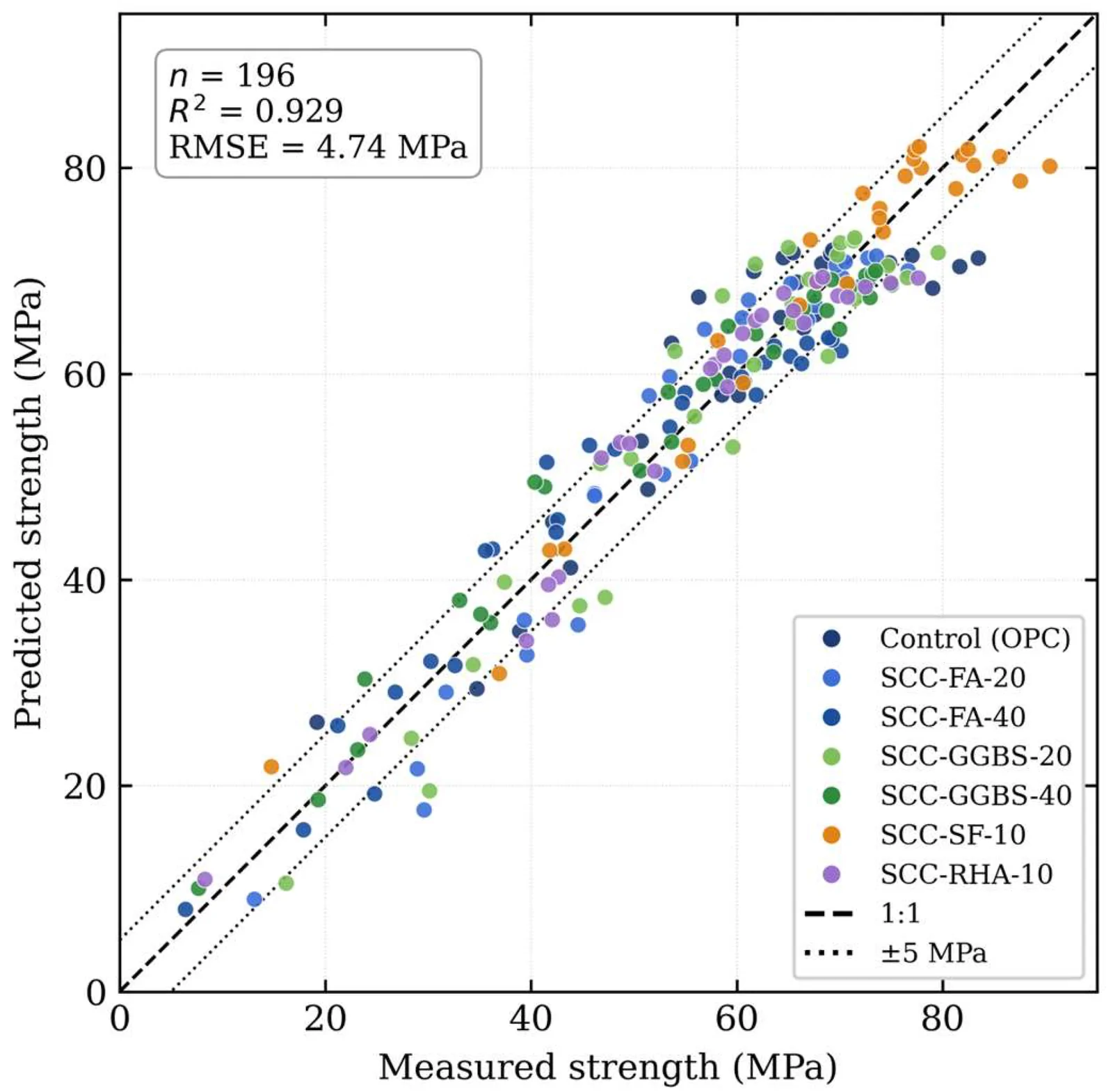
Parity plot of predicted versus observed compressive strength for the proposed temperature-adaptive model (*n* = 196), coloured by material system. The dashed line is 1:1 agreement; dotted lines bound the ±5 MPa envelope. Overall R^2^ = 0.929, RMSE = 4.74 MPa.

Across the full dataset, the model explains 92.9% of the strength variance (R^2^ = 0.929), with RMSE = 4.74 MPa (8.4% of mean strength), a mean absolute error (MAE) of 3.83 MPa, and a mean absolute percentage error (MAPE) of 8.2%—a reasonable level for engineering strength-estimation applications. The residual distribution is essentially symmetric about zero (mean error +0.12 MPa, skewness +0.14) and close to normal (Pearson kurtosis 2.70, Shapiro–Wilk *W* = 0.994, *p* = 0.593), validating least-squares estimation and supporting parametric inference. Figure 4 shows the parity plot: the bulk of the 196 observations fall within the ±5 MPa envelope across the full 6–90 MPa range, clustering tightly around the 1:1 line with no family forming a coherent bias band.

Stratified by material (Table 4), SCC-GGBS-40 achieves the best individual fit (R^2^ = 0.957), followed by SCC-RHA-10 (R^2^ = 0.952). The Control mix shows the lowest fit (R^2^ = 0.836), reflecting the complex crossover behaviour that a linear *Q*(*T*) with positive β_Q_ captures only to first order.

Stratified by curing temperature (Table 5), prediction accuracy within each temperature subset is highest at 50 °C (RMSE = 3.09 MPa) and the explained variance is greatest at 10 °C (R^2^ = 0.942). The 35 °C subset shows the lowest R^2^ (0.863) and the largest-magnitude bias (−2.66 MPa). With every temperature carrying the same *n* = 49 observations, this reflects the position of 35 °C in the transition region—furthest from both the kinetics-controlled and diffusion-controlled asymptotes, and therefore the hardest point for a linear *Q*(*T*) to fit—rather than any sampling imbalance. That R^2^ ≥ 0.863 at every temperature confirms the linear *Q*(*T*) is robust across the experimental range.

### 3.4. How Much Activation-Energy Complexity Does the Data Support?

Because β_Q_ is presented as a physical descriptor, it is essential to establish that the data warrant a temperature-dependent *Q*—and to determine whether they justify one temperature-dependent term in *Q*(*T*), or more. Two nested (i.e., simpler and more complex forms differing from the proposed linear law by exactly one additional term) variants were therefore evaluated on the same dataset and group structure: the ASTM C1074 constant-*Q* model (β_Q_ = 0), and a quadratic form adding γ_Q_(*T* − *T*_ref_)^2^ (one extra parameter per material group). Table 6 compares overall performance and the information criteria (statistical model-selection measures that reward goodness of fit while penalising the number of parameters; lower values indicate the better fit–complexity trade-off [19]), computed as $AIC=N\ln\left(\frac{SSE}{N}\right)+2p$ and $BIC=N\ln\left(\frac{SSE}{N}\right)+p\ln\left(N\right)$, with *p* the total parameter count and *n* = 196.

The comparison shows that the data support exactly one interpretable degree of temperature freedom in *Q*. Adding the single β_Q_ term to the constant-*Q* baseline improves fit (+0.7 percentage points R^2^, −4.8% RMSE) and is favoured by AIC (ΔAIC = +9); the more conservative BIC, which applies a heavier penalty per parameter (ln *N* ≈ 5.3, versus 2 for AIC) to the five additional β_Q_ parameters (one per material group) marginally favours the simpler constant-*Q* form (ΔBIC = −8). Extending to a quadratic *Q*(*T*) is not effective: it modestly improves raw fit only (R^2^ = 0.934), is essentially tied with the linear form under AIC (ΔAIC = −4) and is clearly rejected by BIC (ΔBIC = +12). Hence neither criterion justifies the quadratic form, while between the linear and constant-*Q* forms the verdict depends on how strictly parsimony is weighted: AIC favours the linear model and BIC the constant-*Q* one.

Two considerations that the information criteria cannot capture decide the question in favour of the linear β_Q_ term. The first is interpretability: the constant-*Q* model has no parameter that distinguishes binder chemistry by temperature response, whereas β_Q_ provides exactly the signed fingerprint developed in Section 3.2, at the expense of only one additional parameter per binder system. The second consideration is systematic bias at the lowest and highest curing temperatures: because AIC and BIC summarise performance over the entire dataset, they cannot reveal whether a model is consistently biased within given temperature subsets. Table 7 compares RMSE and bias against the constant-*Q* baseline by temperature. The proposed model lowers RMSE at three of four temperatures and reduces the magnitude of bias where it matters the most: the constant-*Q* model’s cold-weather bias at 10 °C (−1.29 MPa) is essentially eliminated (−0.09 MPa), and that at 50 °C falls from −0.96 to +0.04 MPa. At 35 °C the proposed model is marginally worse in RMSE (+7.4%), reflecting the difficulty of the transition region noted above. The linear β_Q_ is therefore the right operating point: it is the simplest form that is both mechanistically interpretable and effective at the temperature extremes where maturity predictions drive construction decisions. The trade-off between complexity, interpretability and predictive performance should be stated plainly: BIC favours the constant-energy model, and the parameter-uncertainty analysis of Section 3.8 shows that for the plain OPC mixture a constant *Q* is indeed statistically adequate; the linear law earns its additional parameter in the SCM-blended systems, whose temperature sensitivity is statistically significant and whose bias at the temperature extremes it removes.

### 3.5. Independent Data Validation

Generalisability was assessed on an independent literature dataset compiled from two published experimental studies [20,21]. After domain filtering (binder types OPC, FA, GGBS; replacement 0–40%; curing temperature 10–50 °C; age 1–90 days), 120 observations from multiple laboratories, material sources, and procedures were retained. The retained data comprise, from Ref. [20], an OPC control and a 30% GGBS mixture cured at 30, 40 and 50 °C and tested at 1–28 days (18 observations each) and, from Ref. [21], a vibrated OPC control together with 15% and 30% fly-ash mixtures cured at 10, 30, 40 and 50 °C and tested at 1–64 days (28 observations each); a 45% fly-ash series and all 128-day tests fell outside the stated domain limits and were excluded. No kinetic parameter was recalibrated for any external mixture. Most available data mainly contained OPC, fly-ash, and GGBS systems; silica fume and rice husk ash were not significantly represented in the available literature, so their calibrated fingerprints could not be tested externally and rest on the laboratory dataset alone. Because absolute strength is highly sensitive to source-specific properties not captured by the calibration set, validation follows ASTM C1074 practice [1]: the kinetic parameters (λ, *k*, *Q*_0_, β_Q_) are transferred unchanged from Table 3, while the asymptotic strength *S*_u0_ is re-estimated per material group from the external data. This isolates the temperature-history component of prediction error, for which the maturity model is responsible, from the mix-specific absolute scale.

Across the 120 observations, the model explains 88.1% of the strength variance (Table 8), with near-zero mean bias (+0.21 MPa overall; −0.38, +0.58, and +0.60 MPa for OPC, FA, and GGBS). The modest degradation relative to calibration (R^2^ lower by 4.8 percentage points and RMSE higher by 32% relative to calibration) is consistent with the documented behaviour of maturity models from independent material sources [22]. Because the subset sizes are modest, bootstrap 95% confidence intervals were computed for the subset *R*^2^ values by case resampling (2000 draws): [0.65, 0.82] for OPC, [0.85, 0.94] for FA and [0.65, 0.93] for GGBS. The GGBS interval is wide, reflecting its 18 observations, and its point estimate should be read with corresponding caution. The residual degradation is most plausibly attributable to material, testing and curing variability across laboratories rather than systematic model deficiency. The model reproduces strength development for binder families and replacement levels that were not part of the calibration set, which indicates that its temperature-history component generalises reliably.

### 3.6. Residual Diagnostics

**Figure 5:**
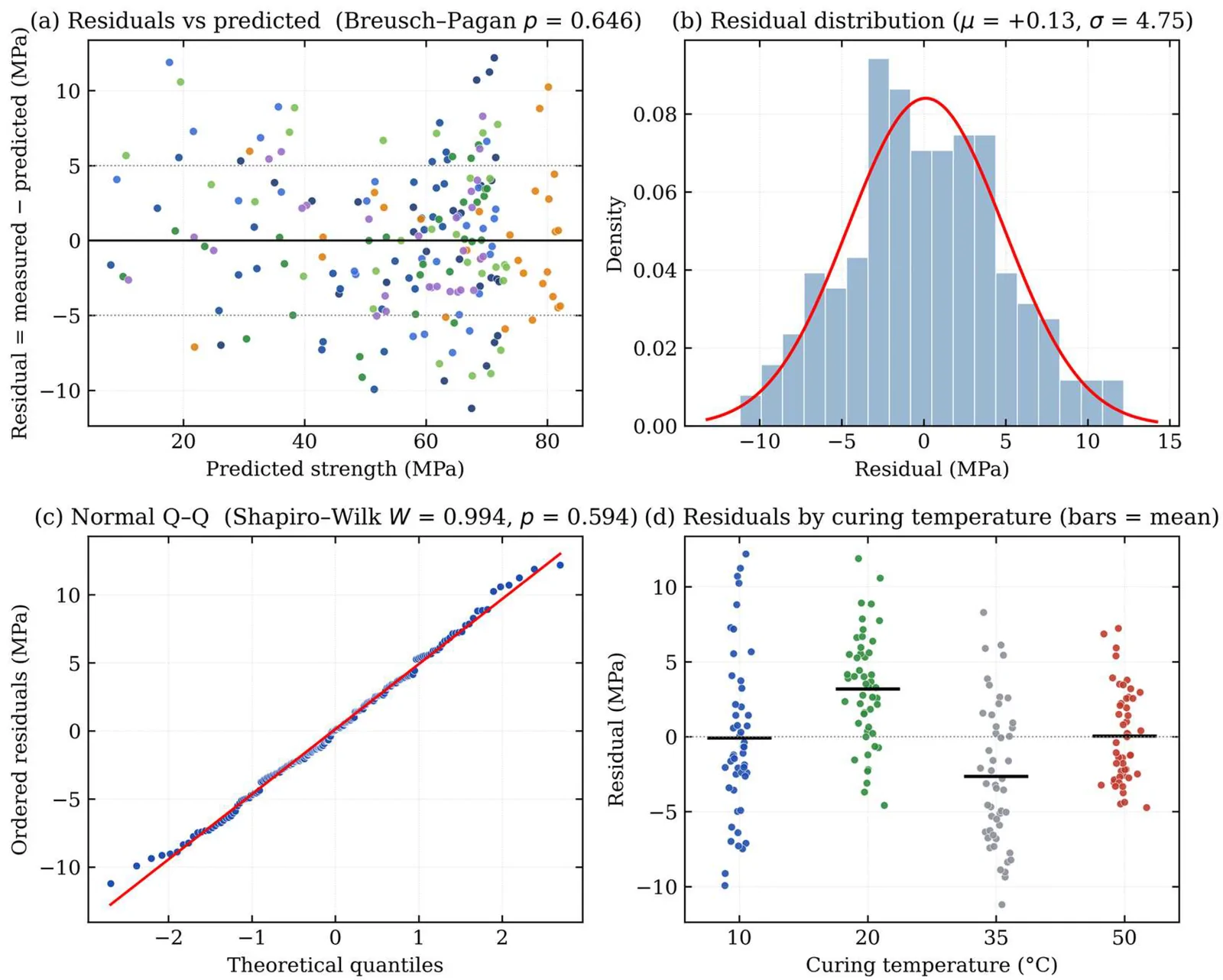
Residual diagnostics for the proposed model (*n* = 196). (**a**) Residuals vs. predicted strength show no systematic trend (Breusch–Pagan *p* = 0.651). (**b**) Residual distribution with overlaid normal density; mean residual ≈ 0, standard deviation = 4.75 MPa. (**c**) Normal Q–Q plot (Shapiro–Wilk *p* = 0.593). (**d**) Residuals stratified by curing temperature; the 35 °C group carries the largest median bias; point colours in panels (**a**,**d**) follow the material-system legend of Figure 4.

Residual analysis on all *n* = 196 observations (Figure 5) supports the model assumptions. The Shapiro–Wilk test (*W* = 0.994, *p* = 0.593 ≫ 0.05) finds residuals indistinguishable from a normal (Gaussian) distribution, with skewness +0.14 and kurtosis 2.70. A Breusch–Pagan test (BP = 0.20, *p* = 0.651 ≫ 0.05) confirms constant error variance across the 6.4–90.4 MPa range, and an ordinary least-squares (OLS) regression of residuals on predicted strength gives a near-zero slope (−0.022), confirming the absence of trend bias.

Seven observations have absolute residuals > 10 MPa (>2.1 standard deviations), concentrated among the Control specimens at cold curing (10 °C), where the model underpredicts late-age strength by up to +12.2 MPa. This indicates that the linear *Q*(*T*) with positive β_Q_ is only a first-order approximation of the OPC crossover: its single positive slope reproduces the average late-age reversal but not the additional curvature seen at 10 °C, so a mixture-specific nonlinearity remains unexplained at that temperature. A quadratic *Q*(*T*) would in principle reduce this residual but, as shown above, is not warranted by either information criterion on the present dataset.

### 3.7. Out-of-Sample Predictive Accuracy

The performance metrics of Section 3.3, Section 3.4 and Section 3.5 are computed on the calibration data and therefore describe in-sample fit; Table 9 quantifies predictive accuracy on unseen data using the protocols of Section 2.4. Under five-fold cross-validation, the model retains *R*^2^ = 0.901 (RMSE = 5.59 MPa, MAE = 4.44 MPa) against the in-sample *R*^2^ = 0.929, a modest and expected degradation indicating that the reported accuracy is not an artefact of in-sample fitting. Temperature transfer is more demanding: withholding an interior temperature gives *R*^2^ = 0.83 (20 °C) and 0.74 (35 °C), whereas withholding a boundary temperature forces extrapolation of the linear *Q*(*T*) beyond the calibrated range and accuracy degrades markedly at 50 °C (*R*^2^ = 0.49). The practical implication is that calibration should span the temperature range of the intended application—precisely the requirement ASTM C1074 already places on maturity calibration [1]—and the model should not be extrapolated beyond it. Replacement-level transfer within the FA and GGBS families is satisfactory, particularly in the 20% → 40% direction (*R*^2^ = 0.87–0.90), supporting the linear dilution term over the calibrated 0–40% range.

### 3.8. Parameter Uncertainty, Identifiability and Prediction Intervals

Table 10 reports bootstrap 95% confidence intervals for the calibrated parameters. Three results qualify the physical reading of β_Q_. First, the negative coefficients of the FA, GGBS and RHA systems are statistically significant—their intervals exclude zero by wide margins—so the kinetics-to-diffusion interpretation is established for the slow-reacting SCM systems. Second, the OPC interval spans zero: the positive point estimate is consistent with the late-age crossover but cannot be distinguished from a constant-*Q* description, in agreement with the preference of BIC for the constant-energy model (Section 3.4); the crossover-based reading of the OPC sign is therefore indicative rather than established. The SF interval also spans zero, consistent with silica fume being the least temperature-sensitive of the four SCMs. Third, for the single-replacement-level SF and RHA mixtures the parameters *S*_u0_ and λ are not separately identifiable—only the combination *S*_u0_ − 0.1λ is constrained by the data—which quantifies the caution stated in Section 3.1 and confirms that the fitted λ of these mixtures must not be transferred to other replacement levels. Parameter correlations show the expected trade-offs (ρ(*Q*_0_, β_Q_) ≈ −0.8 for the Control and FA groups; ρ(*S*_u0_, λ) = +0.92 for the FA family) without pathological confounding among the kinetic parameters. Because each observation is perturbed by the standard error of its three replicate cubes, the measured within-group variability is propagated directly into these intervals, addressing the role of replicate scatter in parameter estimation.

Combining parameter and residual uncertainty yields a 95% prediction interval of approximately ±9.4 MPa for an individual strength estimate, dominated by the residual component. For strength-based decisions such as formwork removal, the corresponding one-sided lower bound (predicted strength minus approximately 7.9 MPa at 95% one-sided confidence) should be used in place of the point prediction, together with the safety factors of normal practice.

## 4. Discussion

### 4.1. Comparison with Prior Variable-Q Models

Table 11 positions the proposed model against established maturity formulations. Freiesleben Hansen and Pedersen [23] introduced the maturity equation with a single constant *Q* and no temperature dependence. Carino and Tank [24] proposed a piecewise-constant *Q*, discontinuous at the regime thresholds. Schindler [3] formulated *Q* as a function of both degree of hydration α and temperature, requiring isothermal calorimetry to obtain α. Kim et al. [25] used a time-varying apparent activation energy *Q* = *E*_0_(*T*)e^−αt^, which is harder to calibrate. The distinguishing feature of the present *Q*(*T*) = *Q*_0_ + β_Q_(*T* − *T*_ref_) is not only that it is the simplest temperature-dependent form in this comparison but also that its temperature-sensitivity parameter is an interpretable, signed descriptor of the binder, fitted from routine strength data and consistent with independent calorimetry.

### 4.2. Practical Implementation and Field Implications

Field deployment requires only a one-time material-characterisation programme followed by routine temperature monitoring. The five parameters (*S*_u0_, λ, *k*, *Q*_0_, β_Q_; four for OPC) are calibrated once per mixture family from isothermal tests at a minimum of three temperatures (e.g., 10, 20, and 35 °C) and several ages. Thereafter, embedded sensors recording *T*(*t*) are the only in situ measurement: equivalent age is obtained by numerical integration of Equation (2), strength is predicted in real time via Equation (1), and a safety factor is applied before authorising operations. For a mixture not previously characterised, β_Q_ is obtained from the same specimen set that ASTM C1074 already prescribes for maturity calibration [1]: (i) cast companion specimens and cure them isothermally at a minimum of three temperatures spanning the expected service range; (ii) test at five or more ages covering early and late strength development; (iii) estimate the parameters of Equations (1)–(3) with the two-stage optimisation of Section 2.3, for which standard open-source routines suffice; and (iv) verify the fit with the residual diagnostics of Section 3.6. Integration into existing practice is direct, because the workflow, instrumentation and specimen requirements are identical to conventional ASTM C1074 calibration; the only change is that the equivalent-age integrand evaluates *Q*(*T*) from Equation (3) instead of a constant value—a minor software modification in commercial maturity systems. The prediction-interval bound of Section 3.8 then supplies the uncertainty margin for strength-based decisions. To make the procedure immediately usable, an interactive single-file calculator implementing Equations (1)–(3) with the calibrated parameters of Table 3—including the prediction-interval bounds—is provided as Supplementary Material File S1.

The bias-reduction result has direct consequences for temperature-sensitive concreting. For the fly-ash family (*Q*_0_ ≈ 48.68 kJ/mol, β_Q_ ≈ −803 J/(mol·K)), the calibrated law gives *Q*(10 °C) ≈ 56.7 kJ/mol—well above the constant *Q*_ASTM_ = 33.5 kJ/mol. The constant value understates how strongly cold curing retards hydration, so the ASTM model accumulates equivalent age too quickly at 10 °C. Reducing its −1.29 MPa cold-weather bias to −0.09 MPa removes a systematic error at the temperature where formwork-removal calls are most timing-critical. Beyond prediction, the sign and magnitude of β_Q_ give the mix designer a direct, comparable read on each binder’s temperature sensitivity: SCM-rich mixtures, with their strongly negative β_Q_, exhibit a less pronounced crossover than plain OPC, which favours their use where elevated placement temperatures are expected. By making strength development in SCM-blended, low-clinker mixes predictable across the field temperature range, the model removes a practical barrier to specifying the high-replacement binders that lower the embodied carbon of concrete.

### 4.3. Embodied-Carbon Context of the Studied Mixtures

The sustainability framing of this work rests on the established role of SCMs in reducing the clinker content of concrete, and it can be quantified for the specific mixtures studied. Applying published cradle-to-gate emission factors—0.912 kg CO_2_e/kg for CEM I, 0.083 for GGBS, 0.008 for fly ash and 0.028 for silica fume [26], and 0.15 for treated RHA including its thermal processing [27]—to the binder masses of the mix designs reported in [9] gives the binder-related embodied carbon in Table 12: cement replacement reduces it by approximately 8–40% relative to the control, reaching −39.7% for SCC-FA-40 and −36.4% for SCC-GGBS-40. These are indicative, factor-based estimates rather than a full life-cycle assessment—system boundaries, transport and allocation choices would refine them—but they quantify the incentive for specifying high-replacement binders in both superstructures and foundations [11,12,13]. The contribution of the present model is complementary: by restoring reliable in-place strength prediction across the field temperature range, it removes a practical barrier—uncertain strength-development timing—to adopting exactly these low-carbon mixtures.

### 4.4. Limitations

Six limitations frame the directions for future work. First, the linear *Q*(*T*) is adequate at 10, 20, and 50 °C but slightly less accurate at the 35 °C transition point (−2.66 MPa bias); a quadratic extension could reduce this mid-range residual but is not supported by AIC or BIC on the present data, and linear extrapolation beyond 10–50 °C should be treated with caution. Second, calibration used isothermal water-tank curing; real structures experience non-isothermal histories (diurnal cycles, internal hydration heat in mass concrete) under which accuracy has not yet been tested, and the saturated-curing assumption (>95% relative humidity, RH) would require moisture-correction terms for dry curing. Third, the calibration covers binary binder systems at single replacement levels; ternary and quaternary blends will need additional data and likely interaction terms. Fourth, SCM coverage is limited to FA, GGBS, SF, and RHA; extension to metakaolin, calcined clay, and higher replacement levels would broaden applicability and further populate the β_Q_ fingerprint library. Fifth, cross-validation shows that predictive accuracy degrades when the model is asked to extrapolate to curing temperatures outside the calibrated range (Section 3.7); calibration must therefore span the intended service temperatures. Sixth, the external validation, although drawn from independent laboratories, covers two experimental programmes with OPC, FA and GGBS binders only, and the GGBS subset is small (n = 18, with a bootstrap 95% confidence interval for *R*^2^ of [0.65, 0.93]); the silica fume and RHA fingerprints therefore rest on the laboratory dataset alone, and broader multi-laboratory validation—including field, non-isothermal curing histories—remains the most important next step.

## 5. Conclusions

This study developed and evaluated a physics-based maturity model with a temperature-adaptive activation energy for predicting compressive-strength development in high-strength SCC incorporating four supplementary cementitious materials. The model combines a hyperbolic strength–maturity relationship, an Arrhenius equivalent age, and a linear activation-energy function *Q*(*T*) = *Q*_0_ + β_Q_(*T* − *T*_ref_), calibrated on 196 consolidated observations (588 cube tests) from seven SCC mixtures cured isothermally at four different temperatures and tested at seven ages. The principal findings are:

- The temperature sensitivity β_Q_ is a signed binder fingerprint. All four SCM systems returned negative β_Q_ (−210 J/(mol·K) for SF, through −636 for GGBS and −803 for FA, to −974 for RHA), confirming a kinetics-to-diffusion transition; the OPC control mix alone returned positive β_Q_ = +101 J/(mol·K), a point estimate whose bootstrap confidence interval spans zero. Bootstrap confidence intervals establish the FA, GGBS and RHA coefficients as significantly negative, indicating that β_Q_ reflects a characteristic of the binder chemistry.
- The model achieved R^2^ = 0.929 and RMSE = 4.74 MPa overall, with R^2^ ≥ 0.836 for every material system and R^2^ ≥ 0.863 at every curing temperature.
- Five-fold cross-validation confirms out-of-sample accuracy (*R*^2^ = 0.901, RMSE = 5.59 MPa). Accuracy degrades when extrapolating to curing temperatures outside the calibrated range, so calibration should span the intended service temperatures, consistent with ASTM C1074 practice. The 95% prediction interval for an individual strength estimate is approximately ±9.4 MPa.
- The data support exactly one interpretable degree of temperature freedom in *Q*. The single β_Q_ term improves on the ASTM C1074 constant-*Q* baseline (+0.7 pp R^2^, −4.8% RMSE) and is favoured by AIC and predictive accuracy; a quadratic extension is warranted by neither AIC nor BIC. The linear form is preferred because it is the simplest that is both mechanistically interpretable and effective at the extreme temperature values.
- Validation on 120 independent literature observations gave R^2^ = 0.881 with a near-zero mean bias (+0.21 MPa), indicating that the calibrated binder fingerprints generalise to independent material sources.
- The chief practical benefit is bias reduction at the temperature extremes—the cold-weather bias of the constant-*Q* model (−1.29 MPa at 10 °C) is essentially eliminated—which directly supports strength-based formwork-removal decisions in temperature-sensitive concreting.

The model requires only four to five parameters per material system together with routine temperature monitoring for field implementation, offering a parsimonious, physically interpretable alternative to constant-*Q* maturity practice for sustainable, SCM-blended self-compacting concrete, and a compact descriptor β_Q_ that characterises binder temperature sensitivity directly from strength data.

## Figures and Tables

**Table 1:** Statistical summary of compressive strength by material system (MPa, *n* = 196). Data from [9].

Material	n	Mean	Std Dev	Min	Max	CV (%)
Control (OPC)	28	60.7	14.7	19.2	83.4	24.2
SCC-FA-20	28	54.8	15.8	13.1	76.6	28.9
SCC-FA-40	28	47.5	17.7	6.4	70.1	37.2
SCC-GGBS-20	28	57.2	16.0	16.2	79.5	28.0
SCC-GGBS-40	28	53.4	19.3	7.7	75.0	36.2
SCC-SF-10	28	68.2	17.1	14.7	90.4	25.0
SCC-RHA-10	28	54.8	16.5	8.3	77.6	30.1
Overall	196	56.7	17.8	6.4	90.4	31.4

**Table 2:** Model parameters, physical meanings, and calibration bounds.

Parameter	Units	Physical Meaning	Lower	Upper
S_u0_	MPa	Ultimate strength at infinite age (for OPC)	50	100
λ	MPa	Strength reduction per unit SCM replacement	0	50
k	day^−1^	Rate constant (early-age strength-gain rate)	0.01	2.00
Q_0_	kJ/mol	Baseline activation energy at 20 °C	10	60
β_Q_	J/(mol·K)	Temperature sensitivity of activation energy	−1000	+500

**Table 3:** Calibrated maturity-model parameters (*n* = 196 dataset).

Material	S_u0_	λ	k	Q_0_	β_Q_	R^2^/RMSE
	**(MPa)**	**(MPa)**	**(day^−1^)**	**(kJ/mol)**	**(J/mol·K)**	**(MPa)**
Control (OPC)	72.65	–	0.681	14.13	+101	0.836/5.95
SCC-FA-20	80.48	40.28	0.323	48.68	−803	0.905/4.88
SCC-FA-40	80.48	40.28	0.323	48.68	−803	0.934/4.54
SCC-GGBS-20	77.25	16.18	0.358	46.72	−636	0.878/5.58
SCC-GGBS-40	77.25	16.18	0.358	46.72	−636	0.957/3.95
SCC-SF-10	82.81	1.74	0.598	33.01	−210	0.940/4.20
SCC-RHA-10	74.54	44.10	0.450	51.77	−974	0.952/3.60
Overall (*n* = 196)						0.929/4.74

**Table 4:** Predictive performance by material system (*n* = 196). FA and GGBS families are fit jointly; metrics are evaluated separately on each subset.

Material	n	R^2^	RMSE (MPa)	MAE (MPa)	MAPE (%)
Control (OPC)	28	0.836	5.95	4.88	8.8
SCC-FA-20	28	0.905	4.88	3.98	9.4
SCC-FA-40	28	0.934	4.54	3.85	9.8
SCC-GGBS-20	28	0.878	5.58	4.67	9.7
SCC-GGBS-40	28	0.957	3.95	3.01	7.2
SCC-SF-10	28	0.940	4.20	3.34	6.2
SCC-RHA-10	28	0.952	3.60	3.06	6.5

**Table 5:** Predictive performance by curing temperature (*n* = 196); bias is the mean of (measured − predicted).

Temperature (°C)	n	R^2^	RMSE (MPa)	MAE (MPa)	Bias (MPa)
10	49	0.942	5.32	4.14	−0.09
20	49	0.925	4.85	4.00	+3.18
35	49	0.863	5.34	4.53	−2.66
50	49	0.930	3.09	2.64	+0.04

**Table 6:** Performance and information-criterion comparison of the three nested Q(T) variants (*n* = 196). Parameter counts are totals across all five material-group fits.

Model	Params	R^2^	RMSE	MAE	MAPE	SSE	AIC	BIC
Temp.-Adaptive (Proposed)	24	0.929	4.74	3.83	8.2	4402	658	737
ASTM C1074 Constant-Q	19	0.922	4.98	3.99	8.8	4856	667	729
Quadratic Q(T)	29	0.934	4.57	3.70	8.0	4101	654	749

Proposed vs. constant-Q: +0.7 pp R^2^, −4.8% RMSE; ΔAIC = +9 (favours proposed), ΔBIC = −8 (favours constant-Q). Proposed vs. quadratic: ΔAIC = −4 (marginally favours quadratic), ΔBIC = +12 (favours proposed).

**Table 7:** RMSE and bias comparison by curing temperature: proposed model vs. ASTM C1074 constant-Q. A negative “Change” value indicates lower RMSE for the proposed model; bias is the mean of (measured − predicted).

Temp. (°C)	n	RMSE Prop.	RMSE ASTM	Change (%)	Bias Prop.	Bias ASTM
10	49	5.32	5.74	−7.3	−0.09	−1.29
20	49	4.85	5.61	−13.6	+3.18	+4.29
35	49	5.34	4.97	+7.4	−2.66	−1.54
50	49	3.09	3.16	−2.5	+0.04	−0.96
Overall	196	4.74	4.98	−4.8	+0.12	+0.12

**Table 8:** External validation on filtered literature data (n = 120). Kinetic parameters transferred from calibration; Su0 re-estimated per material group. Bias = mean (measured − predicted).

Material Type	n	R^2^	RMSE (MPa)	MAE (MPa)	Bias (MPa)
OPC Control mix	46	0.766	7.22	6.28	−0.38
Fly Ash	56	0.907	6.19	5.13	+0.58
GGBS	18	0.861	2.97	2.44	+0.60
Overall	120	0.881	6.26	5.18	+0.21

**Table 9:** Out-of-sample validation of the proposed model: five-fold cross-validation, leave-one-temperature-out and leave-one-replacement-level-out protocols (Section 2.4). Bias is the mean of (measured − predicted).

Protocol	n	*R* ^2^	RMSE (MPa)	Bias (MPa)
Five-fold cross-validation (pooled)	196	0.901	5.59	–
Temperature held out: 10 °C	49	0.751	11.03	−6.36
Temperature held out: 20 °C	49	0.827	7.39	+5.96
Temperature held out: 35 °C	49	0.735	7.42	−4.73
Temperature held out: 50 °C	49	0.492	8.31	+4.23
FA family: 20% → 40% replacement	28	0.868	6.41	−3.17
FA family: 40% → 20% replacement	28	0.737	8.12	+6.23
GGBS family: 20% → 40% replacement	28	0.900	5.99	+0.81
GGBS family: 40% → 20% replacement	28	0.706	8.68	−2.89

**Table 10:** Bootstrap 95% confidence intervals for the calibrated parameters (B = 500, parametric resampling from replicate standard errors). † Interval limited by the calibration bound of −1000 J/(mol·K); the RHA sensitivity should be read as at least this strongly negative. For SF and RHA only the identified combination *S*_u0_ − 0.1λ is reported.

Group	Q_0_ (kJ/mol)	β_Q_ (J/(mol·K))	k (day^−1^)	λ or S_u0_ − 0.1λ (MPa)
Control	13.9 [10.0, 20.3]	+101 [−162, +347]	0.68 [0.61, 0.76]	–
FA	48.8 [45.7, 51.9]	−803 [−966, −660]	0.32 [0.30, 0.34]	λ: 40.0 [33.6, 45.7]
GGBS	46.8 [42.9, 50.5]	−637 [−782, −448]	0.36 [0.34, 0.38]	λ: 16.2 [10.0, 21.9]
SF	33.2 [26.5, 40.0]	−227 [−534, +77]	0.60 [0.55, 0.66]	S_u0_ − 0.1λ: 82.6 [81.3, 83.9]
RHA	51.6 [48.5, 54.1]	−975 [−1000 †, −807]	0.45 [0.42, 0.48]	S_u0_ − 0.1λ: 70.2 [68.8, 71.6]

**Table 11:** Comparison with variable activation-energy models from the prior literature.

Study	Q(T) Formulation	Parameters	Limitation
Freiesleben Hansen & Pedersen (1977) [23]	Constant Q	1	No temperature dependence
Carino & Tank (1992) [24]	Piecewise constant	2–3	Discontinuous at thresholds
Schindler (2004) [3]	Q = f(α, T)	5–7	Requires degree of hydration α
Kim et al. (2001) [25]	Q = E_0_(T)e^−αt^	3	Time-varying, harder to calibrate
This study	Q = Q_0_ + β_Q_(T − T_ref)	2	Simple, signed binder fingerprint, no extra measurements

**Table 12:** Binder-related embodied carbon of the studied mixtures, computed from the mix proportions reported in [9] and the emission factors of [26,27].

Mixture	Binder CO_2_e (kg/m^3^)	Change vs. Control (%)
SCC-R (Control)	469	–
SCC-SF-10	423	−9.7
SCC-FA-20	376	−19.8
SCC-FA-40	283	−39.7
SCC-GGBS-20	384	−18.2
SCC-GGBS-40	298	−36.4
SCC-RHA-10	430	−8.4

## Data Availability

The original contributions presented in this study are included in the article/Supplementary Materials. Further inquiries can be directed to the corresponding author.

## Supplementary Materials

The following supporting information can be downloaded at https://www.mdpi.com/article/10.3390/ma19163462/s1, File S1: interactive temperature-adaptive maturity calculator (standalone HTML file) implementing Eqs. (1)–(3) with the calibrated parameters of Table 3 and the 95% prediction-interval bounds.
