# Supplementary material for: Temperature-Adaptive Activation Energy for Maturity-Based Strength Prediction of Sustainable, SCM-Blended Self-Compacting Concrete
Source: Materials (Basel). 2026 Aug 14;19(16):3462. doi: 10.3390/ma19163462 (PMC13514605; doi:10.3390/ma19163462)
Supplement: Supplementary file 1 [file materials-19-03462-s001.zip › materials-4473777-Supplementary Material File S1.html]

Supplementary Material S1 — Temperature-adaptive maturity calculator


# Supplementary Material S1 — Interactive temperature-adaptive maturity calculator

Companion to: Aldawish, Kulasegaram, Almutlaqah, Alshahrani, *Temperature-Adaptive Activation Energy for
Maturity-Based Strength Prediction of Sustainable, SCM-Blended Self-Compacting Concrete* (Materials,
manuscript materials-4473777). Implements Eqs. (1)–(3) with the calibrated parameters of Table 3;
no other model is used. Runs entirely in the browser.

Mixture (Table 3)

Curing temperature (°C): 20

Age (days): 28

Predicted compressive strength: –

–

–

**Scope and use.** Valid for isothermal, saturated curing within the calibrated envelope
(10–50 °C, 1–90 days); do not extrapolate outside it (Section 3.7 of the paper). The 95% prediction
interval (±9.4 MPa) combines parameter and residual uncertainty (Section 3.8); for strength-based
decisions such as formwork removal use the one-sided 95% lower bound (prediction −7.9 MPa) together with
the safety factors of normal practice. For non-isothermal field histories, integrate Eq. (2) over the
recorded temperature record. Parameters for SF-10 and RHA-10 are valid at their tested replacement levels
only (Section 3.8).
